# Encapsulation of Hydrocortisone and Mesalazine in Zein Microparticles

**DOI:** 10.3390/pharmaceutics5020277

**Published:** 2013-05-10

**Authors:** Esther T. L. Lau, Steven J. Giddings, Salmaan G. Mohammed, Paul Dubois, Stuart K. Johnson, Roger A. Stanley, Peter J. Halley, Kathryn J. Steadman

**Affiliations:** 1 School of Pharmacy, the University of Queensland, Brisbane, QLD 4072, Australia; E-Mails: e.lau@uq.edu.au (E.T.L.L.); stevengiddings@hotmail.co.uk (S.J.G.); salsgmuk@googlemail.com (S.G.M.); 2 Department of Pharmacy, King's College London, London SE1 8WA, UK; 3 Food Science and Technology Program, Curtin Health Innovation Research Institute, Curtin University, Perth, WA 6102, Australia; E-Mails: p.dubois@curtin.edu.au (P.D.); s.johnson@curtin.edu.au (S.K.J.); 4 Centre for Nutrition and Food Sciences, Queensland Alliance for Agriculture and Food Innovation, The University of Queensland, Brisbane, QLD 4072, Australia; E-Mail: r.stanley@uq.edu.au; 5 Australian Institute for Bioengineering and Nanotechnology, and School of Chemical Engineering, The University of Queensland, Brisbane, QLD 4072, Australia; E-Mail: p.halley@uq.edu.au

**Keywords:** maize, microparticles, protein, drug loading, *in vitro* digestibility, electrophoresis

## Abstract

Zein was investigated for use as an oral-drug delivery system by loading prednisolone into zein microparticles using coacervation. To investigate the adaptability of this method to other drugs, zein microparticles were loaded with hydrocortisone, which is structurally related to prednisolone; or mesalazine, which is structurally different having a smaller LogP and ionizable functional groups. Investigations into the *in vitro* digestibility, and the electrophoretic profile of zein, and zein microparticles were conducted to shed further insight on using this protein as a drug delivery system. Hydrocortisone loading into zein microparticles was comparable with that reported for prednisolone, but mesalazine loading was highly variable. Depending on the starting quantities of hydrocortisone and zein, the average amount of microparticles equivalent to 4 mg hydrocortisone, (a clinically used dose), ranged from 60–115 mg, which is realistic and practical for oral dosing. Comparatively, an average of 2.5 g of microparticles was required to deliver 250 mg of mesalazine (a clinically used dose), so alternate encapsulation methods that can produce higher and more precise mesalazine loading are required. *In vitro* protein digestibility revealed that zein microparticles were more resistant to digestion compared to the zein raw material, and that individual zein peptides are not preferentially coacervated into the microparticles. In combination, these results suggest that there is potential to formulate a delivery system based on zein microparticles made using specific subunits of zein that is more resistant to digestion as starting material, to deliver drugs to the lower gastrointestinal tract.

## 1. Introduction

Hydrocortisone (a corticosteroid) and mesalazine are commonly used in the management of inflammatory bowel disease (IBD). The two main types of IBD are ulcerative colitis (UC) and Crohn’s Disease (CD). Pharmacological management depends on the severity, type of IBD, and the overall goal of treatment [1,2,3].

Corticosteroids are used for their anti-inflammatory effects to induce remission during active UC or CD, or when UC becomes refractory to first-line treatment [1]. These corticosteroids can be delivered intravenously, orally as controlled-release tablets, or locally as rectal foams, suppositories or enemas [2]. Corticosteroids are not used to maintain remission due to dose-dependent side effects [1,2]. Delivering corticosteroids by using a colon-targeted delivery system may mean that smaller doses can be used for a shorter duration of time, which in turn would reduce the dose-related side effects.

Mesalazine has a different mode of action from corticosteroids and is used to induce remission in both UC and CD of mild to moderate severity [3]. It also maintains remission in UC, but this use in CD is controversial [3]. Mesalazine is thought to exert its anti-inflammatory effects locally in the large intestine [2,3]. It is commonly administered via the oral route with controlled-release tablets or granules [2]. One of the strategies used to target the delivery of mesalazine to the large intestine is through the use of pro-drugs, which are inactive when administered, but undergo transformation *in vivo* to become active [3]. In mesalazine pro-drugs, mesalazine is linked to a carrier molecule via an azo-bond. The azo-bond is cleaved in the large intestine by the microbial enzyme azoreductase, which in combination with the reducing environment of the large intestine, leads to release of the active drug molecule from its carrier [3,4,5,6,7]. While these mesalazine pro-drugs are used commercially, one problem with their use is the dosing regimen. Depending on the mesalazine pro-drug formulation, doses in excess of 4 g daily in four divided doses may be used, with side effects more prevalent with higher doses [2,3]. Hence, using an alternative method that targets mesalazine delivery to the large intestine may reduce the frequency of dosing required, and reduce the large doses used, thereby reducing dose related side effects.

Zein is a protein extracted from corn (*Zea mays*), and has previously been investigated for its potential as an oral drug delivery system. It is of interest as a drug delivery system as it has a “generally recognized as safe” food use status [8], and is reportedly resistant to digestion in the stomach [9,10]. Zein has been investigated for use as a tablet excipient [11], and formulated into microparticles to control the release of bioactive agents such as essential oils [12], lysozyme [13], and prednisolone [14] in conditions simulating the human digestive tract. Zein microparticles have also been used to encapsulate abamectin, protecting it from photo-degradation [15]. This photo-stability imparted by encapsulation within zein microparticles would be advantageous for mesalazine because it is light sensitive [16].

One method used to formulate zein microparticles is coacervation, also known as phase separation [10,12,14,17]. Coacervation involves dispersing the core material in a solution of the coating material. The microparticles are formed when the coating material desolvates, coacervating around the core material. Zein microparticles were successfully loaded with prednisolone (a corticosteroid) using a coacervation method [14]. Prednisolone loading and loading efficiency were improved by varying the starting quantities of zein and prednisolone. Additionally, more vigorous and extensive agitation by a vortex mixer compared to a magnetic stirrer increased drug loading and loading efficiency [14].

This present study aims to investigate the formulation of zein microparticles loaded with hydrocortisone compared to loading with mesalazine. Hydrocortisone was selected as it is structurally similar to prednisolone. This was to determine if the methods used for formulating zein-prednisolone microparticles could be applied to a structurally similar drug. Mesalazine was selected as it is structurally different, and has characteristics unlike prednisolone and hydrocortisone. By comparison, mesalazine is more soluble in water [18,19], and has ionizable functional groups whereas prednisolone and hydrocortisone do not. Mesalazine was therefore used to determine if the microparticle formulation method could successfully be applied to drugs with different structures and characteristics. Zein microparticles loaded with either hydrocortisone or mesalazine were formulated using an optimized method previously reported for prednisolone [14]. The drug loading and loading efficiency were compared. To further characterize the zein microparticles as a potential drug delivery system, the protein content, *in vitro* protein digestibility, and protein electrophoretic profile were determined and compared to the zein raw material.

## 2. Experimental Section

### 2.1. Microparticle Formulation

Zein microparticles encapsulating either hydrocortisone (≥98%, Sigma Aldrich, St. Louis, MO, USA), or mesalazine (≥99%, Sigma Aldrich, St Louis, MO, USA) were formulated using coacervation from 70% (*v*/*v*) ethanol [14]. The core material was dissolved/dispersed in 12 mL of 70% (*v*/*v*) ethanol. Zein (Sigma Aldrich, St Louis, MO, USA) was added to the hydrocortisone solution or mesalazine dispersion, and mixed using a vortex mixer (Zx^3^, Velp Scientifica, Usmate, Italy) for 1 min. Water (MilliQ; 8 mL) was added and the formulation was vortex mixed for a further 2 min to produce microparticles, and these were subsequently freeze-dried. Microparticles formulated without the core material, or without zein were used as the controls. Samples with mesalazine were wrapped in aluminum foil and prepared in a dimmed room to protect them from light. Each formulation of microparticles was made in triplicate to assess the variation between each replicate. Reagents were of analytical and HPLC grades.

### 2.2. Analysis of Drug Loading in Microparticles and Encapsulation Efficiency

#### 2.2.1. Hydrocortisone Microparticles

**Figure 1:**
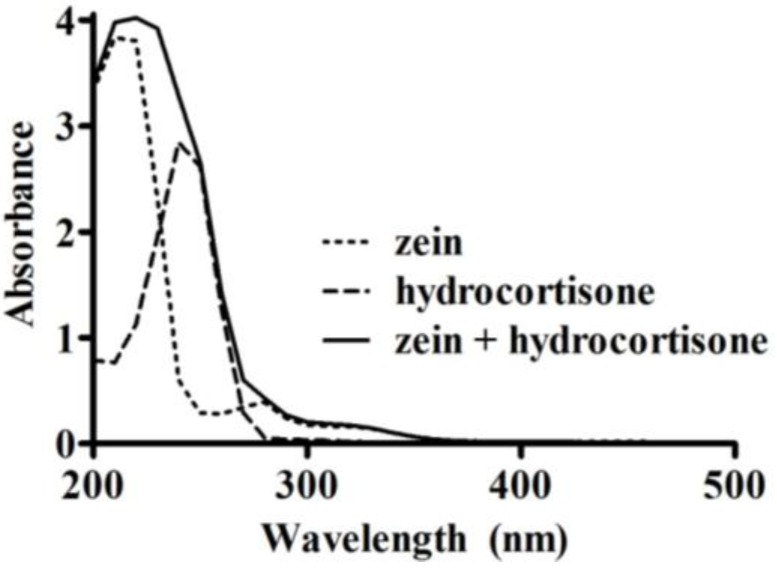
UV absorbance spectra (200–500 nm) of zein (0.5 mg/mL), hydrocortisone (51 µg/mL), and a mixture of zein (0.5 mg/mL) with hydrocortisone (51 µg/mL) in 70% (*v*/*v*) ethanol.

Freeze-dried microparticles (10 mg) were washed by vortex mixing for 20 s with 1 mL of ethyl acetate to remove any non-encapsulated hydrocortisone. The mixture was centrifuged to recover the ethyl acetate supernatant. The wash was repeated three times, after which the microparticles and ethyl acetate supernatants were dried using a centrifugal vacuum evaporator (Savant RVT400, Thermo Fisher Scientific, Waltham, MA, USA). For each sample, the dried microparticles and the hydrocortisone extracted by the ethyl acetate were dissolved in 70% (*v*/*v*) ethanol. The amount of hydrocortisone present in the microparticles, and the amount washed out by ethyl acetate were measured using a UV spectrophotometer (U-1900, Hitachi, Tokyo, Japan) at 254 nm. This wavelength was determined based on an UV scan (200–500 nm, at 400 nm/min) of zein (0.5 mg/mL), hydrocortisone (51 µg/mL), and a combination of zein (0.5 mg/mL) and hydrocortisone (51 µg/mL) in 70% (*v*/*v*) ethanol. A wavelength of 254 nm was appropriate to measure the absorbance of hydrocortisone as there was minimal interference from zein (Figure 1). Three 10 mg aliquots were measured from each microparticle sample to assess variability in drug loading and loading efficiency within each replicate. Calibration curves in the range of 0–33 µg/mL were used to quantify the amount of hydrocortisone, with measurements made in triplicate for each sample.

#### 2.2.2. Mesalazine Microparticles

**Figure 2:**
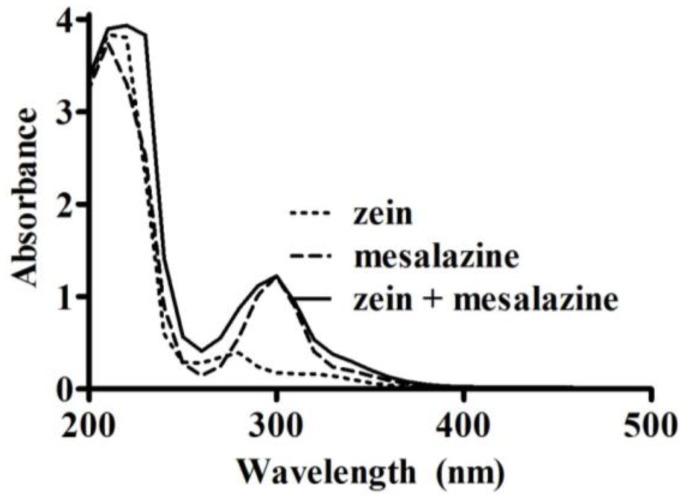
UV absorbance spectra (200–500 nm) of zein (0.5 mg/mL), mesalazine (51 µg/mL), and a mixture of zein (0.5 mg/mL) with mesalazine (51 µg/mL) in 70% (*v*/*v*) ethanol.

The method for measuring loading and loading efficiency was adapted for mesalazine because it is reported as “*practically insoluble*” in ethyl acetate [16]. Mesalazine solubility was assessed in different solvents (Table 1) to determine an appropriate substitute for ethyl acetate. The solvent pH was adjusted using 1 M HCl. An excess of mesalazine was added to 5 mL of solvent, protected from light and left to mix on a suspension mixer at 10 °C. After 22 h, it was allowed to re-equilibrate for 2 h at room temperature. Excess mesalazine was removed following centrifugation, and the supernatant was analyzed using a UV spectrophotometer. Zein had minimal interference on the absorbance of mesalazine at 297 nm (Figure 2). This wavelength was determined based on an UV scan (200–500 nm, at 400 nm/min) of zein (0.5 mg/mL), mesalazine (51 µg/mL), and a combination of zein (0.5 mg/mL) and mesalazine (51 µg/mL) in 70% (*v*/*v*) ethanol. The amount of mesalazine was quantified on the UV spectrophotometer at 297 nm using calibration curves in the range of 0–59 µg/mL.

**Table 1:** Mesalazine solubility (mg/mL) in different solvents at varying pH and temperature.

Solvent	pH	Temperature (°C)	Solubility (mg/mL)
70% ( *v*/*v*) ethanol	6.5	25	0.81
70% ( *v*/*v*) ethanol	6.5	37	1.40
70% ( *v*/*v*) ethanol	4.5	25	0.81
0.01 M HCl	2	25	0.89
0.1 M HCl	1	25	1.50
Distilled water	6.5	25	0.80

Mesalazine loading and loading efficiency was analyzed using the method described for hydrocortisone microparticles, except 1 mL of ethyl acetate was replaced with 10 mL of water and the process took place in a dimmed room. Mesalazine solubility in 70% (*v*/*v*) ethanol at 25 °C, and at pH 4.5 was comparable to that of distilled water (pH 6.5) (Table 1). Its solubility increased with an increase in temperature to 37 °C. The observed solubility of mesalazine in distilled water was in agreement with that reported in literature, being 0.84 mg/mL at 25 °C, and 1.41 mg/mL at 37 °C [20]. Reducing the pH to 1 also improved solubility [20]. However, the macrostructure of zein microparticles we formulated at pH 1 appeared hard and crystalline, as opposed to the soft coarse powder formed using a more neutral pH of 6.5 although the structure of zein is reportedly not affected in environments above pH 1 [21]. Therefore, distilled water at 25 °C was selected to replace ethyl acetate in the wash step due to convenience and ease of use, and because water dissolved mesalazine but not zein. The water supernatant from the washes and microparticle pellets were freeze-dried. The amount of mesalazine was quantified as above.

### 2.3. Calculating Drug Loading into Microparticles and Encapsulation Efficiency

Drug loading, *i.e.*, the amount of drug contained within the microparticles, and loading efficiency, *i.e.*, the amount of encapsulated drug as a proportion of the amount added initially, were calculated using the following two equations.

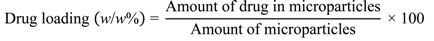
(1)

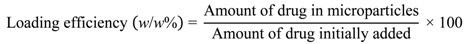
(2)

### 2.4. Particle Size Distribution

The particle size distribution of the freeze dried microparticles was determined via laser diffraction using a Mastersizer 2000E and a small volume sample dispersion unit (Malvern Instruments Ltd., Malvern, UK). Freeze dried microparticles (50 mg) were added to 50 mL of 40% ethanol, and sonicated for 30 min until the protein was well dispersed. The refractive index of 1.45 was used for zein and 1.36 for ethanol. The obscuration range was between 10% and 20% with a residual of less than 1%. Particle size was measured in duplicate for each sample.

### 2.5. Protein Determination

Nitrogen content of zein and zein microparticles (empty) was determined in triplicate using the Dumas combustion method (AACC Standard Method 46–30) [22], then protein content was calculated as nitrogen × 6.25.

### 2.6. *In Vitro* Protein Digestibility

Protein digestibility was measured using an *in vitro* digestibility assay for cereal proteins which included incubating the samples with pepsin at pH 2, then quantifying the nitrogen content of the undigested fraction [23]. Incubation time was limited to 1 h to prevent complete digestion of all samples [24], to allow subtle differences between the digestibility of the zein compared to the resulting microparticles to be identified. Triplicate samples (100 mg) were weighed into pre-dried and pre-weighed 50 mL centrifuge tubes, and vortex mixed for 1 min with 35 mL of freshly prepared pepsin solution (1.5 mg/mL in 0.1 M phosphate buffer at pH 2). A reagent blank tube was also prepared. All tubes were incubated for 60 min at 37 °C in a water bath (OLS200, Grant Instruments, Cambridge, UK) shaking at 100 cycles/min. After 60 min, 2 M NaOH (2 mL) was added to each tube, then centrifuged at 4900*g* at room temperature for 20 min. The supernatant containing the digested protein was discarded, while the pellets of undigested protein were washed with 20 mL of 0.1 M phosphate buffer (pH 7) to remove entrapped digested protein, and centrifuged as above. This washing and centrifuging was then repeated, and the resulting pellets were dried overnight in an oven at 60 °C. The weight of the dried pellet was calculated from the final weight of the dried pellet and tube. The nitrogen content of a known mass of the dried pellet was determined by the Dumas combustion method as above and protein digestibility was calculated based on nitrogen content data using the following equation:

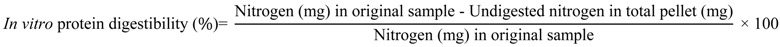
(3)

### 2.7. Reducing Sodium Dodecyl Sulfate-Polyacrylamide Gel Electrophoresis (SDS-PAGE)

Samples (5 mg) were dissolved in 1 mL of 0.0125 M borate buffer (pH 10) containing 1% SDS and 2% 2-mercaptoethanol by vortex mixing for 5 min, then centrifuged for 5 min at 12,900*g* at room temperature [25,26,27]. The protein extract supernatants (5 µL) were mixed with 10 µL of loading buffer (NuPAGE LDS, Invitrogen, Carlsbad, CA, USA), heated at 95 °C for 5 min, then 10 µL of each sample solution was loaded in a precast 4%–15% polyacrylamide SDS-PAGE gel (TGX Mini Gels, BioRad Laboratories Inc, Hercules, CA, USA). The gel was electrophoresed in Tris/Glycine buffer (BioRad Laboratories Inc, Hercules, CA, USA) at 200 V for 25–30 min at room temperature using a Mini Protean system (BioRad Laboratories Inc, Hercules, CA, USA) until the pre-stained molecular weight marker proteins were clearly separated and the dye was within 1 cm of the bottom of the gel. The gel was then washed three times, for 5 min each, in deionized water, stained with Coomasie Stain (BioRad Laboratories Inc, Hercules, CA, USA) for 1.5 h, then washed with several changes of deionized water for 2 h. Gels were scanned using a BioRad BioScan Image analyzer (BioRad Laboratories Inc., Hercules, USA).

### 2.8. Statistical Analysis

One-way ANOVA with a Bonferroni *post hoc* test were used to compare the mean drug loading, loading efficiency, and the amount of microparticles required to encapsulate a clinically relevant dose of each drug (hydrocortisone or mesalazine). Mean values for protein content, and *in vitro* protein digestibility were compared by applying an unpaired *t*-test with GraphPad Prism 5.04 (GraphPad Software, San Diego, CA, USA). The level of significance was set as *p* < 0.05, and the coefficient of variation (%CV) was calculated to compare the variance between and within replicates of samples.

## 3. Results and Discussion

### 3.1. Drug Loading and Loading Efficiency

**Figure 3:**
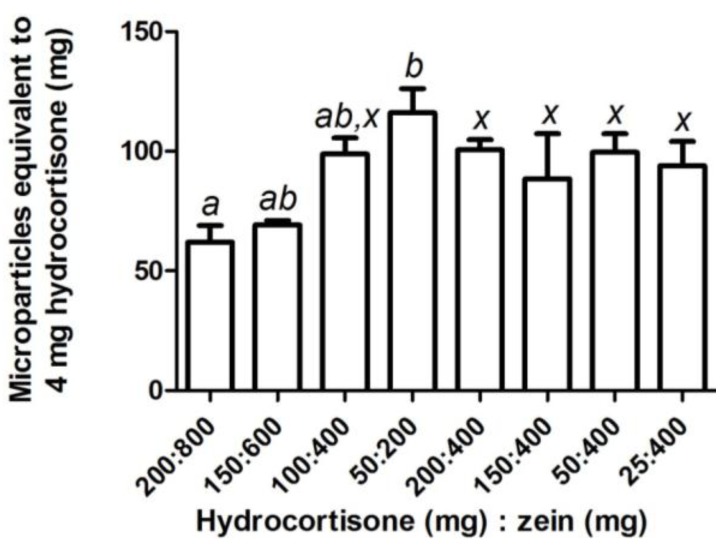
The amount of zein-hydrocortisone microparticles (mean ± SEM; *n* = 3) equivalent to a clinically used dose of hydrocortisone (4 mg) for samples prepared using various combinations of hydrocortisone (25–200 mg) and zein (200–800 mg). One-way factorial ANOVA with a *post hoc* Bonferroni test was used to compare selected means for varying quantities with constant ratio of zein and mesalazine (*a–b*), and a constant quantity of zein with varying mesalazine quantity (*x*). The level of significance was set at *p* < 0.05 and means that were not significantly different were assigned the same letter.

Hydrocortisone and mesalazine could successfully be loaded into zein microparticles. Increasing the starting quantities of zein and hydrocortisone at a constant ratio increased hydrocortisone loading and loading efficiency (Table 2). Drug loading was unchanged while loading efficiency increased when 400 mg of zein was formulated with decreasing quantities of hydrocortisone; most notably when less than 100 mg of hydrocortisone was used (Table 2). Depending on the starting quantities of hydrocortisone and zein, the average amount of microparticles equivalent to 4 mg hydrocortisone, a clinically used dose, ranged from 60 to 115 mg (Figure 3). Hydrocortisone, which closely resembles prednisolone in structure, showed similar trends in terms of drug loading and loading efficiency as previously reported for prednisolone [14]. Particle size for prednisolone microparticles [14] and hydrocortisone microparticles (*d*(*v*,0.1) = 0.442 µm, *d*(*v*,0.5) = 1.011 µm, *d*(*v*,0.9) = 11.305 µm, *D*[3,2] = 0.919 µm, *D*[4,3] = 4.163 µm) were also very similar. These similarities may be explained by the similar properties that prednisolone and hydrocortisone possess due to their related structures (Table 3).

**Table 2:** Drug loading and loading efficiency (mean ± SEM) into zein microparticles formulated using various combinations of zein and hydrocortisone, with the coefficient of variation (%CV) for within and between replicates. One-way factorial ANOVA with a *post hoc* Bonferroni multiple comparisons test was used to compare selected means (*p* < 0.05).

Zein (mg)	Drug (mg)	Ratio	%CV			Total mean ± SEM	Total %CV
			Rep 1	Rep 2	Rep 3		
*Hydrocortisone loading (w/w%)*							
800	200	4	7.71	15.43	3.01	5.41 ± 0.60 *^a^*	19
600	150	4	7.41	2.97	1.90	4.72 ± 0.05 *^ab^*	2
200	50	4	5.66	3.98	21.67	2.84 ± 0.25 *^b^*	16
400	200	2	1.07	7.36	6.37	2.69 ± 0.08 *^x^*	5
400	150	2.7	5.25	4.87	7.98	3.68 ± 0.86 *^x^*	40
400	100	4	10.11	1.95	9.44	3.31 ± 0.20 *^b,x^*	11
400	50	8	3.11	8.16	8.49	3.66 ± 0.28 *^x^*	13
400	25	16	2.18	25.09	11.25	3.77 ± 0.44 *^x^*	20
*Hydrocortisone loading efficiency (w/w%)*							
800	200	4	6.66	12.64	3.42	33.13 ± 4.55 *^A^*	24
600	150	4	4.28	2.80	2.38	27.47 ± 2.28 *^AB^*	14
200	50	4	8.31	1.56	27.00	16.03 ± 1.38 *^B^*	15
400	200	2	4.40	10.47	11.18	9.44 ± 0.17 *^X^*	3
400	150	2.7	5.53	1.48	19.01	14.54 ± 2.63 *^X^*	31
400	100	4	26.98	7.51	19.66	20.66 ± 0.39 *^B,X^*	3
400	50	8	5.16	6.05	4.27	40.55 ± 1.64 *^Y^*	7
400	25	16	1.98	3.78	1.27	84.18 ± 2.40 *^Z^*	5

Means were grouped as follows for statistical analysis, with the means that were not significantly different at *p* < 0.05 assigned the same letter: varying quantities with constant ratio of zein and hydrocortisone (*a–b*, *A–B*), constant quantity of zein with varying hydrocortisone quantity (*x*, *X–Z*).

**Table 3:** The molecular structure, pKa, LogP, aqueous solubility, and solubility in ethanol of prednisolone, hydrocortisone and mesalazine.

	Prednisolone	Hydrocortisone	Mesalazine
Molecular structure	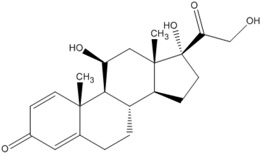	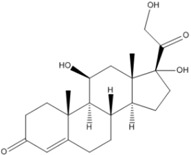	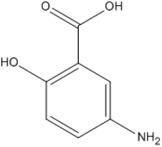
pKa	-	-	2.15, 7.10, 12.30 [20]
Log P	1.62 [28]	1.61 [28]	0.64 [18]
Aqueous solubility at 25 °C	0.273 mg/mL [19]	0.311 mg/mL [19]	1.85 mg/mL [18]
Solubility in absolute ethanol	24.3 mg/mL at 25 °C [19]	14.7 mg/mL at 25 °C [19]	0.324 mg/mL at 24 °C [29]

**Figure 4:**
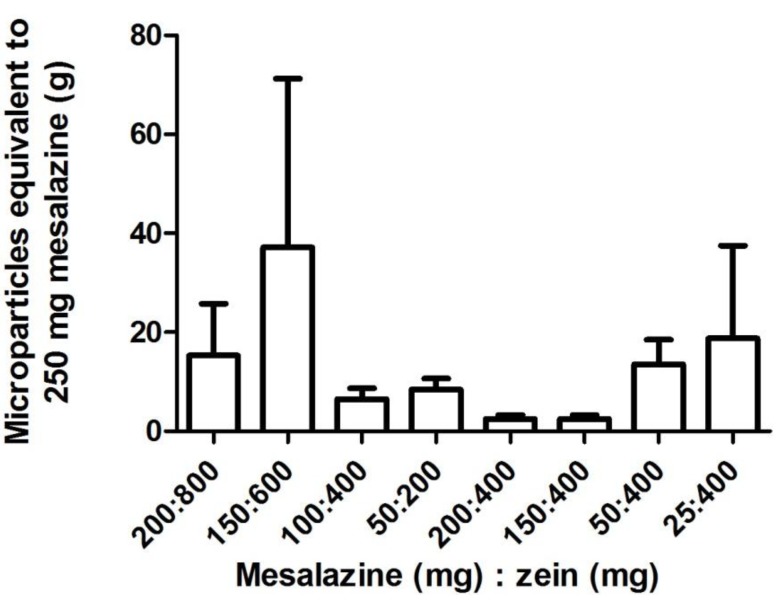
The amount of zein-mesalazine microparticles (mean ± SEM; *n* = 3) equivalent to a clinically used dose of mesalazine (250 mg) for samples prepared using various combinations of mesalazine (25–200 mg) and zein (200–800 mg). One-way factorial ANOVA with a *post hoc* Bonferroni test was used to compare selected means (varying quantities with constant ratio of zein and mesalazine, and a constant quantity of zein with varying mesalazine quantity). The means were not significantly different at *p* < 0.05.

Conversely, mesalazine, which is structurally very different and has different properties compared to prednisolone and hydrocortisone (Table 3), did not produce similar drug loading and loading efficiency trends. General trends were visible in mesalazine loading and loading efficiency but the extremely high variation between the replicates meant that a statistical difference was not observed (Table 4). When the ratio of drug to protein was kept constant, the mesalazine loading and loading efficiency into the zein microparticles appeared to be most improved with the larger starting quantities of 200 mg mesalazine and 800 mg zein (Table 4). A constant amount of zein (400 mg) with increasing quantities of mesalazine appeared to increase drug loading and loading efficiency. However, these trends lack statistical significance due to large differences between the three replicates prepared for each zein-mesalazine combination (Table 4). Nonetheless, the loading efficiency for the combination of 400 mg zein and 150 mg mesalazine was significantly higher than the combination with 25 mg mesalazine. Using the combination with the highest drug loading (*i.e.*, 400 mg zein and 150 mg mesalazine), an average of approximately 2.5 g of microparticles was equivalent to 250 mg of mesalazine, which is a clinically used dose (Figure 4). A large gelatin capsule (size 000) that is administered orally has a capacity of 822–1644 mg depending on the density of the contents used to fill the capsule [30], meaning that the amount of microparticles required to deliver a clinically relevant dose of mesalazine is 1.5 to 3-fold more than the capacity of a size 000 gelatin capsule. Particle size of mesalazine microparticles (*d*(*v*,0.1) = 1.381 µm, *d*(*v*,0.5) = 5.047 µm, *d*(*v*,0.9) = 12.730 µm, *D*[3,2] = 3.322 µm, *D*[4,3] = 6.154 µm) indicated that they were larger in size than the hydrocortisone, and prednisolone microparticles.

**Table 4:** Drug loading and loading efficiency (mean ± SEM) into zein microparticles formulated using various combinations of zein and mesalazine, with the coefficient of variation (%CV) for within and between replicates. One-way factorial ANOVA with a *post hoc* Bonferroni multiple comparisons test was used to compare selected means (*p* < 0.05).

Zein (mg)	Drug (mg)	Ratio	%CV			Total mean ± SEM	Total %CV
			Rep 1	Rep 2	Rep 3		
*Mesalazine loading (w/w%)*							
800	200	4	6.79	14.67	10.02	4.84 ± 3.38 *^a^*	121
600	150	4	8.61	26.28	4.21	4.66 ± 2.34 *^a^*	87
200	50	4	5.98	13.9	42.02	4.09 ± 1.54 *^a^*	65
400	200	2	19.05	5.91	1.57	8.02 ± 1.87 *^x^*	41
400	150	2.7	12.18	7.69	11.04	9.70 ± 2.84 *^x^*	51
400	100	4	1.58	4.25	2.41	2.91 ± 0.98 *^a,x^*	59
400	50	8	5.90	28.59	19.78	2.21 ± 0.77 *^x^*	60
400	25	16	17.96	ND	ND	0.14 ± 0.14 *^x^*	ND
*Mesalazine loading efficiency (w/w%)*							
800	200	4	7.60	12.78	3.38	33.65 ± 15.90 *^A^*	82
600	150	4	11.39	19.19	11.90	23.37 ± 13.33 *^A^*	99
200	50	4	4.77	12.80	37.06	24.71 ± 4.23 *^A^*	30
400	200	2	8.08	3.02	3.19	40.08 ± 4.77 *^X,Y^*	21
400	150	2.7	6.00	4.31	8.59	61.69 ± 9.61 *^X^*	27
400	100	4	2.74	12.66	22.48	20.97 ± 4.75 *^A,XY^*	39
400	50	8	2.12	28.00	11.20	21.57 ± 7.79 *^XY^*	63
400	25	16	15.49	ND	ND	2.73 ± 2.73 *^Y^*	ND

Means were grouped as follows for statistical analysis, with the means that were not significantly different at *p* < 0.05 assigned the same letter: varying quantities with constant ratio of zein and mesalazine (*a*, *A*), constant quantity of zein with varying mesalazine quantity (*x*, *X–Y*). ND: %CV could not be calculated as no mesalazine loading was observed.

The overall %CV in the zein microparticles formed with mesalazine (loading 41%–121%, loading efficiency 21%–99%; Table 4), was generally larger than that of hydrocortisone (loading 2%–40%, loading efficiency 3%–31%; Table 2), indicating a lack of reproducibility in loading mesalazine into the zein microparticles using this formulation method. Drug loading and loading efficiency of both hydrocortisone and mesalazine was more reproducible within each sample, than between samples, as the %CV within each set of replicates (three sub-samples were measured for each set of microparticles that were prepared) was generally smaller than that between replicates. The more variable mesalazine loading into zein microparticles, compared to hydrocortisone, might be explained by the different chemical structures. Hydrocortisone is a steroid consisting of a five-membered ring, and three six-membered rings with no ionizable groups. Mesalazine consists of a single ring structure with three ionizable groups (carboxyl, amine and hydroxyl groups). The pKa of these ionizable groups are 2.15, 7.10 and 12.30 respectively [20], so at a neutral pH, the carboxyl group would be ionized with a negative charge, the amine group partially ionized with a positive charge, and the hydroxyl group would be unionized. Hence, during the formulation of the microparticles in 70% (*v*/*v*) ethanol, some of the mesalazine molecules would exist as zwitterions, *i.e.*, ionized with both a positive and negative charge. The presence of both these charges on some mesalazine molecules may have interfered with the inter-molecular interactions involved in the formation of zein microparticles during the coacervation process. Zein is amphiphilic in character [31], and self-arranges into micelle-like aggregates [32]. In the presence of other particles, zein forms small aggregates with the hydrophobic zein moieties facing the hydrophobic particles when water is added; and aggregates with the hydrophilic zein moieties facing the hydrophilic particles when ethanol is added [31,32]. During the formation of zein-hydrocortisone microparticles, the hydrophobic nature of hydrocortisone would have helped to promote the formation of these micelle-like structures by interacting with the hydrophobic zein moieties. Furthermore, hydrocortisone lacks ionizable groups to interfere with the formation of these micelle-like structures. Conversely, when the comparatively less hydrophobic mesalazine was loaded, the negatively charged mesalazine and those present as zwitterions may have caused zein to use different molecular arrangements [31] to accommodate the differently charged mesalazine molecules. This, in conjunction with the different mesalazine charges, gives rise to inter- and intra-molecular repulsions during the assembly of these micelle-like structures, causing erratic and non-reproducible loading. Mesalazine is not considered a hydrophilic compound *per se*, but its less hydrophobic and charged nature compared to prednisolone and hydrocortisone may mean that adding ethanol to the zein-mesalazine dispersion (as opposed to water described herein) might result in improved loading. This is because ethanol will cause the hydrophobic moieties in zein to be orientated towards the solvent, and the hydrophilic moieties to be oriented toward the mesalazine molecule, which may facilitate the formation of zein-meslazine microparticles [32]. Preliminary results from microparticles formulated by adding pure ethanol to the zein-mesalazine dispersion (400 mg zein and 100 mg mesalazine) also resulted in variable, albeit higher mesalazine loading and loading efficiency. Mesalazine loading was 29.34% ± 7.12% (%CV = 42.04%), and loading efficiency was 53.03% ± 3.10% (%CV = 10.14); mean ± SEM; unpublished data. Hence, future studies could further investigate this method of formulating zein-mesalazine microparticles.

The variability observed between the replicates of mesalazine loaded microparticles might also be due to the method of agitation used during the coacervation process. More vigorous and extensive mixing provided by a vortex mixer improved drug loading, as was noted with loading prednisolone into zein microparticles [14], but the use of a vortex mixer may have contributed to the variability. This is because there was a brief time lapse where the mixture could not be agitated between removing and replacing the lid of the centrifuge tube when water was added to induce coacervation. This temporary stasis in the mixture may have allowed mesalazine to settle in solution, such that when microparticles were formed with coacervation, varied amounts of mesalazine were encapsulated. Hence, an alternative method of mixing that provides continuous agitation, and more energy to the solution (e.g., a magnetic stirrer or a sonicator probe), may in fact help to keep the mesalazine particles evenly dispersed and suspended while zein is deposited around it.

Compared to hydrocortisone and prednisolone, mesalazine is more soluble in water and less soluble in 70% (*v*/*v*) ethanol (Table 1, Table 5) [19,20]. As such, higher drug loading values were expected for mesalazine-loaded microparticles than hydrocortisone-loaded microparticles, as saturating the aqueous phase with the core material reportedly allows for improved encapsulation [33,34]. Similarly, the mesalazine being dispersed rather than dissolved in the 70% (*v*/*v*) ethanol was also thought to improve drug loading as the dispersed drug particles would form a core on which the zein could coacervate. However, large variability in drug loading and loading efficiency between replicates of the zein-mesalazine microparticles makes this comparison difficult.

The solubility of mesalazine is improved at the extremes of pH, where one ionized species dominates [20]. While such pH environments may not be ideal for formulating zein microparticles, an alternative coating material that is compatible at such a pH may still be used to form microparticles with mesalazine. The presence of only one charge, together with an increase in solubility may assist mesalazine loading into microparticles with more reproducibility using a simple coacervation process like the one described herein. Mesalazine may also be loaded into microparticles using a method similar to complex coacervation, which involves the use of charged coating materials [35,36].

The sensitivity of mesalazine to heat and light makes it difficult to improve its solubility using conventional methods (e.g*.*, increasing temperature). Given these constraints, it is difficult to reproducibly formulate zein-mesalazine microparticles with high drug loading, high loading efficiency, and with precision using a simple coacervation method. As such, another method of encapsulation that can accommodate the restrictions imposed by the low solubility of mesalazine in ethanol, along with light and heat sensitivities, may have more success in formulating zein microparticles loaded with higher levels of mesalazine. Methods such as spray chilling may be applicable. Spray chilling is similar to spray drying except it is suitable for heat sensitive material because cooled or chilled air are used [37,38].

### 3.2. Protein Content and *in Vitro* Protein Digestibility

The protein content of the zein raw material was consistent with previous reports that it was not 100% pure protein [39,40]. Neither the zein raw material nor the resulting empty zein microparticles that were not loaded with drug were 100% pure protein, with the protein content of the zein starting material being significantly higher than the empty zein microparticles (Table 5). The empty zein microparticles were of even lower protein content, possibly due to artifacts of the microparticle formulation process, but these components were not quantified in the present study. The *in vitro* protein digestibility of the empty zein microparticles was significantly lower than the zein raw material. The amphiphilic nature of zein makes it thermodynamically favorable for the zein to self-assemble into colloid/micelle like structures [31,41]. Hence, this more stable structure of the microparticles may be more resistant to digestion compared to the zein raw material. Empty zein microparticles may have potential as an oral delivery system that targets the lower gastrointestinal tract e.g., as tablet coating, or using empty zein microparticles as a tablet excipient.

**Table 5:** Protein content and *in vitro* protein digestibility of zein and empty zein microparticles. Values are mean ± standard deviation (*n* = 3). Means within a column that were not significantly different at *p* < 0.05 were assigned the same letter: protein content (*A*,*B*), and *in vitro* protein digestibility (*a*,*b*).

Sample	Protein content (g/100 g)	*In vitro* protein digestibility (%)
Zein (raw material)	85.4 ± 0.4 ^*A*^	93.3 ± 2.2 ^*a*^
Zein microparticles (empty)	82.9 ± 1.0 ^*B*^	50.3 ± 5.8 ^*b*^

### 3.3. Reducing Sodium Dodecyl Sulfate-Polyacrylamide Gel Electrophoresis

**Figure 5:**
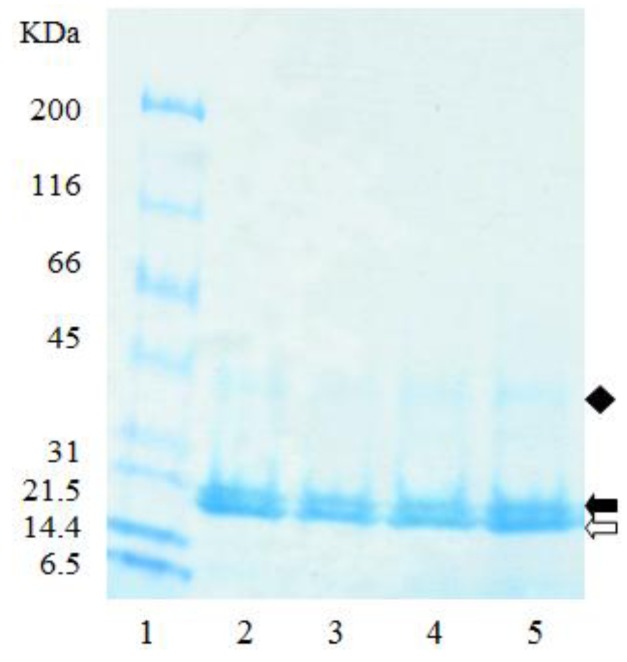
SDS-PAGE of zein and empty zein microparticles extracted with borate buffer under reducing conditions. (**1**) MW standards; (**2**), (**3**) zein; (**4**), (**5**) empty zein microparticles. Clear arrow, and solid arrow are peptides c. 20 kDa; solid diamond are peptides c. 40 kDa.

The electrophoretic profile of the zein starting material, and the resulting empty microparticles were similar, with both having two major bands present at 20 kDa, and a third at 40 kDa (Figure 5), suggesting that individual zein peptides were not preferentially coacervated into the microparticles (Figure 5). The two major bands present at 20 kDa are likely to represent the major subunits of α-zein (21–25 kDa) which account for approximately 80% of total zein [42]. Other minor protein classes are β-zein (10% of the total zein, 17–18 kDa), and γ-zein (10% of the total zein, approximately 27 kDa) [42]. Accordingly, this suggests that the band at 40 kDa is neither β-zein nor γ-zein, but a dimer of the α-zein peptides. While γ-zein was not visible on the gel (Figure 5), likely due to its low concentrations, it is known to be most resistant of the zein peptides to digestion [43]. Therefore, zein protein from maize varieties with higher levels of γ-zein may be useful for the development of microparticles for drug delivery to the lower gastrointestinal tract.

## 4. Conclusions

Hydrocortisone and mesalazine can successfully be formulated into microparticles with zein using coacervation. The amount of microparticles required for a clinical dose of hydrocortisone is realistic and practical. If this zein delivery system for hydrocortisone is resistant to the digestive processes of the stomach and small intestine, it may offer benefits over the commercially available immediate-release hydrocortisone tablets. However, given the large doses of mesalazine used clinically, the amount of microparticles required for a dose of mesalazine is not practical. This, in conjunction with the lack of reproducibility in mesalazine loading into the zein microparticles using this formulation method, precludes its usefulness in a clinical setting. Hence, loading mesalazine into zein requires further investigation to produce higher drug loading yields with more precision. Further investigations include assessing an alternate method of encapsulation, or a modified version of the methods described herein, e.g., increasing the ethanol concentration to induce coacervation and using a magnetic stirrer for a continuous source of agitation. A method that can reproducibly formulate zein microparticles with high mesalazine loading means that zein may have more success as an orally delivered, delayed or controlled-release drug delivery system for mesalazine. *In vitro* protein digestibility and electrophoretic profiles of zein microparticles (compared to the raw material) suggest there is potential to formulate a delivery system to deliver drugs to the lower gastrointestinal tract, based on zein microparticles made using specific subunits of zein that is more resistant to digestion.
